# Study and interest of cellular load in respiratory samples for the optimization of molecular virological diagnosis in clinical practice

**DOI:** 10.1186/s12879-016-1730-9

**Published:** 2016-08-09

**Authors:** Paul Bonnin, Fabien Miszczak, Nathalie Kin, Cecile Resa, Julia Dina, Stephanie Gouarin, Florent Viron, Remy Morello, Astrid Vabret

**Affiliations:** 1 Normandie University, Caen, France; 2 EA 4655-U2RM, UNICAEN, F-14032 Caen, France; 3 Department of Virology, CHU de Caen, F-14033 Caen, France; 4 Statistics and Clinical Research Department, CHU de Caen, F-14033 Caen, France

**Keywords:** Nasal swab, Nasal aspirates, Respiratory virus, Cellular load, Cell quantification, Acute respiratory infections, Virological diagnosis

## Abstract

**Background:**

Respiratory viral diagnosis of upper respiratory tract infections has largely developed through multiplex molecular techniques. Although the sensitivity of different types of upper respiratory tract samples seems to be correlated to the number of sampled cells, this link remains largely unexplored.

**Methods:**

Our study included 800 upper respiratory tract specimens of which 400 negative and 400 positive for viral detection in multiplex PCR. All samples were selected and matched for age in these 2 groups. For the positive group, samples were selected for the detected viral species.

**Results:**

Among the factors influencing the cellularity were the type of sample (*p* < 0.0001); patient age (*p* < 0.001); viral positive or negative nature of the sample (*p* = 0.002); and, for the positive samples, the number of viral targets detected (0.004 < *p* < 0.049) and viral species.

**Conclusion:**

The cellular load of upper respiratory samples is multifactorial and occurs for many in the sensitivity of molecular detection. However it was not possible to determine a minimum cellularity threshold allowing molecular viral detection. The differences according to the type of virus remain to be studied on a larger scale.

## Background

Acute respiratory infections (ARI) are a major cause of morbidity and mortality in children under five, the elderly, and vulnerable patients [1]. Upper respiratory tract infection incidence is estimated at 7–8 per year in children under 4, and at 2–4 in adults [2].

Most respiratory viruses belong to five different viral families (*Para- and Ortho-myxoviridae, Picornaviridae, Coronaviridae, Adenoviridae*), and include 14 viral species, defining what is called the “respiratory panel” [3]. Some of them have a high potential of emergence and can cause pandemics. Although some viruses have been associated with particular diseases (respiratory syncytial virus and bronchiolitis, parainfluenza virus 3 and laryngitis, rhinovirus and common cold, influenza virus and flu syndrome), there is no evidence for a clinical specificity, and only the virological diagnosis provides an accurate identification of the ARI [4, 5].

Detection of respiratory viruses is of little interest in general practice, in that the infection does not present a risk of severity for the patient. However, virological confirmation of ARI is needed in severe clinical presentations, requiring hospitalization in intensive care units and occurring in vulnerable subjects [4, 6]. The goal of early virological diagnosis would be an optimization of patient care, which could lead to reduction in length of hospital stay, a saving of antibiotics, and complementary examinations [7].

Virological tests allow for the establishment of accurate diagnosis of infection, assessment of evolving risks (bacterial infection, acute respiratory distress syndrome), and the establishment of measures to limit its spread (isolation, wearing gloves and masks).

Pandemics of Severe Acute Respiratory Syndrome (SARS, 2002-2003) and influenza A-H1N1 (2009) lead to the development of molecular biological techniques applied to virological diagnosis, mainly based on PCR (polymerase chain reaction). Performances of molecular methods in respiratory virology are so significant that they have replaced conventional techniques (culture, detection of viral antigens) as a reference method [8–11].

Multiplex PCR techniques are particularly suited to medical diagnosis because they can detect multiple viral targets in the same time, avoiding the virologist a selection of viral targets to search. There are now many commercial kits for the detection of a range of 12 to 15 respiratory viruses and some intracellular bacteria [6, 7, 12, 13]. Molecular techniques (real-time PCR) also make it possible to achieve a semi quantification of the viral molecular material present in the sample, giving additional information about the respiratory viral load (interest in therapeutic monitoring and infection transmission risk) [10]. A normalized viral load can be obtained by adding a cell quantification step.

The primary site for replication of respiratory viruses is the ciliated airway epithelium. The sample must be taken as soon as possible after the onset of symptoms. This is usually a nasal swab or nasopharyngeal aspiration (especially realized in children under 2) [14]. These samples are easily accessible and especially adapted to upper ARI. If a rich cell collection appears to be an important prerequisite for the quality of respiratory viral diagnosis, there is currently no information on a possible cellularity threshold that would validate the result of the viral molecular detection.

The main objective of this work is the study of cellularity in 800 respiratory specimens previously characterized virologically. The results should help to define the concept of “cellular richness” and determine the factors that influence it.

## Methods

### Patients and samples

Eight hundred respiratory samples were included in this study. All were collected between September 1, 2010 and February 21, 2013 in different departments of the University Hospital of Caen (France), and immediately sent to the virology department for a respiratory viral diagnosis. Respiratory samples were divided into 604 nasal swabs corresponding to nasopharynx sampling (posterior nares), collected on universal viral transport medium (UTM) and 196 nasal aspirates. After receipt in the laboratory, each sample underwent a pre-analytical step including division into 3 aliquots: one was immediately used for the viral detection and the other two were stored frozen at -80 °C. One of its two frozen fractions was used for this study. This complementary diagnostic study was then conducted on residual clinical specimens, in French law, the right to use the end of the samples is written in the code of public health : Code de la santé publique – Article L1211-2.

These 800 aliquots were selected in the laboratory samples bank according to their results in virological diagnosis: 400 Positive and 400 Negative for molecular detection of respiratory panel using the RespiFinder^®^Smart_22_Fast technique (Pathofinder, Maastricht, Netherlands). This kit allows for the detection of 15 RNA and 2 DNA viral targets and 4 intracellular bacterial targets in respiratory specimens (Tables 1 and 2). A total of 4 age groups reflecting the distribution observed in practice in the laboratory were indicated as follows: Infants (age < 2; 33 %; *n* = 264), Children (aged from 2 to 15 ; 33 %; *n* = 264), Adults (aged from 15 to 65; 21.5 %; *n* = 172) and Elderly (age ≥ 65; 12.5 %; *n* = 100). Each group is composed of half positive and half negative in molecular viral detection, and so as to be matched for age. Within the group of positive samples, the distribution of detected viral species was modeled on that observed in the routine activity of the laboratory (Tables 1 and 2).

**Table 1:** Distribution of detected viruses in the 400 positive samples, according to the age groups constituted (% of detection among the group)

Viral species	Groups			
	Infants *n* = 132	Children *n* = 132	Adults *n* = 86	Elderly *n* = 50
HBoV	2.2	3	0	0
hMPV	5.3	3.8	5.8	12
PIV 1-4	12.9	6	5.8	4
AdV	13.6	16.7	3.5	0
RSV A/B	20.5	10.6	8.1	18
HCoV 229E, NL63, OC43, HKU1	21.2	15.9	13.9	22
Flu A/B	9.8	26.5	40.7	18
RhV/EV	43.2	35.6	30.2	28
% viral co-detection	29	18	8	2

*HBoV* human bocavirus, *hMPV* human metapneumovirus, *PIV* parainfluenza virus, *AdV* adenovirus, *RSV* human respiratory syncytial virus, *HCoV* human coronavirus, *Flu* influenzae virus, *RhV/EV*, rhino/entero virus

**Table 2:** Distribution of nasal aspirates among samples, positive of negative, and between age groups. Distribution was random at inclusion of samples in the study

	Positive samples	Negative samples
Infants	43/132	43/132
Children	45/132	55/132
Adults	9/86	1/86
Elderly	0/50	0/50
Total	97	99

### Nucleic acid extraction

After thawing the samples, the extraction of nucleic acids was performed using the PLC QIAsymphony® (QIAGEN, Hilden, Germany). The extraction was performed with 200 μL of sample using QIAsymphony_DSP_virus/pathogen_minikit® (QIAGEN, Hilden, Germany) and the “complex_200V6_DSP_Respi” program. Nucleic acids were eluted in 140 μl of elution buffer and stored at 4 °C before being analyzed by PCR within 24 h.

### Cellular quantification

Cell quantification was achieved by amplification and detection of a human household gene in real-time PCR (hypoxanthine phosphoribosyl transferase-1) [15, 16]. Cell quantification was performed on a LightCycler 480 II® platform (Roche, Meylan, France). The reaction mixture included 15 μl of amplification premix CELL_Control r-Gene® (Argene/Biomerieux, Lyon, France) and 10 μl of nucleic acid extract. Each manipulation included two negative controls: one undergoing all the analytical steps, called EC (extraction control) and one introduced prior to the PCR reaction, called “negative control”. Cell quantification standard (QS1: 5 × 10^4^ cells/PCR and QS2: 5 × 10^3^ cells/PCR) was ready to use in the kit. An external standard curve was performed using these two standards and 2 additional dilutions, respectively containing 5 × 10^2^ and 50 cells/PCR. The reading of the results was carried out directly from the PLC software, which displays the Ct values (cycle threshold) obtained and the corresponding number of cells/PCR-reaction (i.e. 10 μl of extract). This number was converted to “cells/ml” and the final results were expressed in logarithmic scale (log10/ml). The quantification kit performances were verified in our laboratory by quantifying MRC5 cells (human embryonic fibroblasts) of known concentration (RD-Biothech, Besancon, France). A range of 11 2-fold serial dilutions was made from initial MRC5 cell suspension in viral transport medium (UTM). The nucleic acids were extracted from these 12 cell suspensions, and cell quantification reaction was carried out under the same conditions as for the respiratory samples.

### Data analysis

Descriptive statistics were used to show the characteristics of the different variables. Quantitative variables were described using means and standard deviation. Qualitative variables were described using frequencies and percentages. The relationships between qualitative variables were studied using the Chi-square test or Fisher’s exact tests. The ANOVA was used to compare the means of quantitative variables in two or more independent groups with the Bonferroni post hoc test. The relationship between two quantitative variables was assessed using the Spearman correlation coefficient (ρ). To look for a diagnostic threshold to divide positive and negative samples, a Receiver Operating Characteristic curve (ROC curve) was used.

All the tests were two-tailed and their level of significance (p) was defined as *p* < 0.05. IBM®-SPSS® 22.0 for Windows® was the statistical software used.

## Results

### Quantification kit performances in laboratory

The 2-fold serial dilution range from initial MRC5 suspension had expected cellularity values between 6 Log/ml (concentration given by the manufacturer) and 2.69 Log/ml (last dilution). The measured cellularity values were consistent with those expected for the concentrations between 6 and 3.59 Log. The last 3 dilutions deviated more than 0.4 Log of the expected value, corresponding to concentrations between 3.29 and 2.69 log (Table 3). The mean deviation from expected values was 0.34 Log/ml. Regarding our samples population, cell quantification was negative (no HPRT-1 DNA detection) for 8 of the 400 positive and for 16 of the the 400 negative in viral detection.

**Table 3:** Results of cellular quantification on MRC5 cell suspension dilution range (11 2-fold serial dilutions). The mean deviation from expected values was 0.34 Log/ml. Cellularity values were consistent with those expected for concentrations between 6 and 3.59 log

Expected cellularity Log/ml	Measured cellularity Log/ml	Deviation (absolute value, Log/ml)
6	5.66	0.34
5.70	5.66	0.04
5.40	5.54	0.14
5.10	4.99	0.11
4.80	4.92	0.12
4.49	4.58	0.08
4.19	3.96	0.23
3.89	3.53	0.37
3.59	3.19	0.40
3.29	2.72	0.57
2.99	2.16	0.83
2.69	1.84	0.85

### Comparative study of sample “swabs” and “nasal aspirates”

**Fig. 1:**
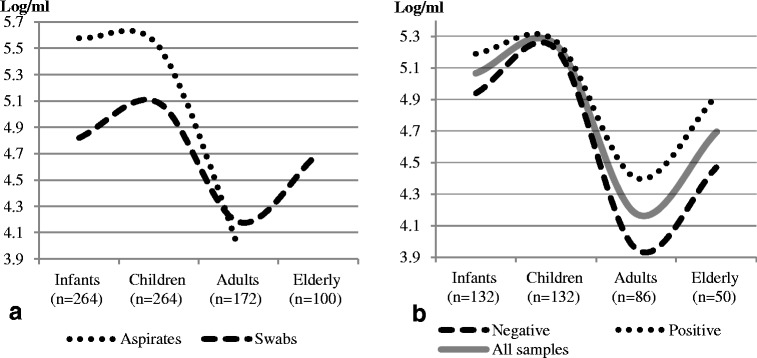
Average cellularity of respiratory specimens depending on the sample type and age group. **a** Nasal aspirate was the sample type that provides, on average, more epithelial cells for patients under the age of 15 (5.70 Log/ml for aspirates versus 4.96 Log/ml for swabs). **b** Although having the same shape, the average cellularity curve of positive samples was always located above the negatives

Among the 800 cell-quantified respiratory samples, 196 were nasal aspirates and 604 were nasal swabs. Cellularity was on average 5.46 (+/- 1.15) Log/ml for aspirates and 4.70 (+/- 1.34) Log/ml for swabs (p <0.0001). For patients under the age of 15, nasal aspirate was the type of sample that provided more epithelial cells (5.70 +/- 1.11 Log/ml for aspirates versus 4.96 +/- 1.05 Log/ml for swabs); in Adults (15-65 yo), the nasal swab provided greater cell concentration (4.18 +/- 1.69 Log/ml for swabs versus 3.99 +/- 1.08 Log/ml for the aspirates); no nasal aspirates were found in the ederly group. These results are presented in Fig. 1a.

### Comparison of cellularity of samples in different age groups

For patients under the age of 15, age did not significantly influence the cell concentration (*p* = 0.377), unlike the type of sample. After 15 y.o., age had a significant influence on the sample cellularity whatever the sample type. The Children group had the richest samples (5.25 +/- 1.08 Log/ml), followed by samples of the Infants group (5.07 +/- 1.12 Log/ml) and the Elderly group (4.70 +/- 1.36 Log/ml), and finally the Adults sample group (4.17 +/- 1.66 Log/ml). These results are presented in Table 4.

**Table 4:** Comparison of the average cellularity of samples taken from different age groups: all samples (*AS*), positive (*P*) in viral detection and negative (*N*) in viral detection (*ns* not significant)

Cellularity (Log/ml):		Infants	Children	Adults	Elderly
for	AS	5.07	5.25	4.17	4.7
	P	5.19	5.27	4.4	4.92
	N	4.94	5.22	3.95	4.47
Infants	AS		ns	*p* < 0.0001	*p* < 0.001
	P		ns	*p* < 0.001	ns
	N		ns	*p* < 0.001	*p* = 0.018
Children	AS	ns		*p* < 0.0001	*p* < 0.0001
	P	ns		*p* < 0.001	ns
	N	ns		*p* < 0.001	*p* = 0.002
Adults	AS	*p* < 0.0001	*p* < 0.0001		*p* = 0.011
	P	*p* < 0.001	*p* < 0.001		*p* = 0.015
	N	*p* < 0.001	*p* < 0.001		*p* = 0.029
Elderly	AS	*p* < 0.001	*p* < 0.0001	*p* = 0.011	
	P	ns	ns	*p* = 0.015	
	N	*p* = 0.018	*p* = 0.002	*p* = 0.029	

Regarding nasal swabs only, average cellularity were not significantly different between the age groups except for children compared with adults (*p* <0.001).

### Comparison of cellularity among the Positive (*n* = 400) and Negative (*n* = 400) samples in viral detection

**Fig. 2:**
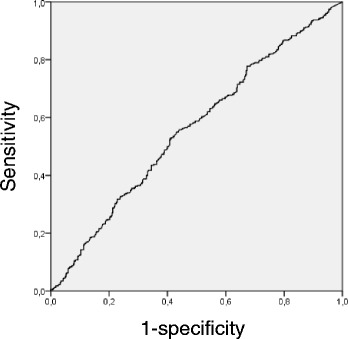
ROC curve (Receiver operating characteristic). Samples cellularity is not a predictive marker of positive or negative result of molecular virus detection

As the subjects were matched for age, the age distribution is identical in the two groups Positive and Negative (*p* = 0.996). These two groups are comparable, as expected. The average cellularity was 5.01 (+/- 1.25) Log/ml for the Positive group and 4.76 (+/- 1.41) Log/ml for the Negative group. This difference was significant (*p* = 0.002). The results of comparison between the age groups according to the result of the viral detection (Positive or Negative) are presented in Fig. 1b. Within a single age group (Infants, Children, Adults, Elderly), the differences between positive and negative samples were not significant (*p* = 0.134, *p* = 0.552, *p* = 0.074 and *p* = 0.098 respectively). Based on the results of the comparison between Positive and Negative samples, a ROC (receiver operating characteristic) curve was performed. No minimum cellularity threshold could be defined for molecular viral detection (Fig. 2).

### Study of samples cellularity according to the viral species detected

**Fig. 3:**
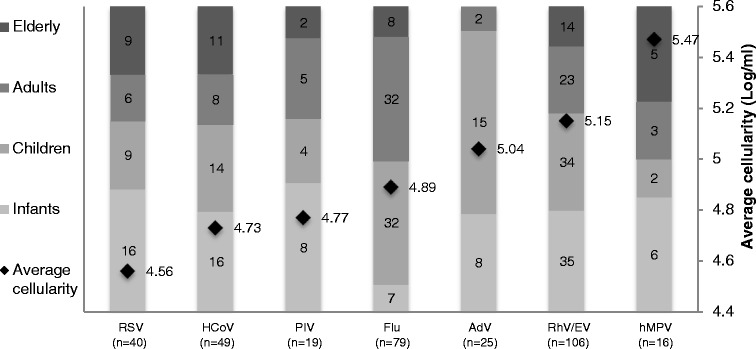
Average cellularity of samples according to the single-detected viral species. The numbers in the columns gives the distribution of samples on the age groups. The average cellularity for a single-detected virus is indicated by the diamond (see right ordinate)

The average cellularity was determined for each viral species detected in the positive samples for a single virus (*n* = 338/400). The 62 viral co-detection samples were excluded. The results were as follow: RSV = 4.56 (+/- 1.27) Log/ml (*n* = 40); HCoV = 4.73 (+/- 1.45) Log/ml (*n* = 49); PIV 1-4 = 4.77 (+/- 1.37) Log/ml (*n* = 19); Flu A-B = 4.89 (+/- 1.29) Log/ml (*n* = 79); AdV = 5.04 (+/- 0.94) Log/ml (*n* = 25); RhV/EV = 5.15 (+/- 1.20) Log/ml (*n* = 106); hMPV = 5.47 (+/- 0.85) Log/ml (*n* = 16) (Fig. 3). There is a significant difference of cellularity between RSV and RhV/EV positive samples (*p* = 0.012), between RSV and hMPV positive samples (*p* = 0.015), and between HCoV and hMPV positive samples (*p* = 0.041).

### Samples with viral co-detections (2 viruses or more)

Among the 800 selected samples, viral detection was negative in 400, 338 were positive for 1 viral target, 58 were positive for 2 targets and 4 were positive for 3 targets. The average cellularity was 4.76 (+/- 1.41) Log/ml, 4.95 (+/- 1.26) Log/ml, 5.30 (+/- 1.17) Log/ml, and 6.19 (+/- 0.21) Log/ml for these 4 groups respectively. The average cellularity in Negative samples was significantly lower than in cases of mono (*p* = 0.049), bi (*p* = 0.004) or tri-detection (*p* = 0.032). A significant tendency was observed between positive samples for one viral target and those positive for 2 or 3 virus (*p* = 0.064), this trend was confirmed by a Spearman correlation (ρ = 1) indicating a strong correlation between sample cellularity and the number of viruses detected.

## Discussion

Molecular detection, including multiplex techniques, is currently the gold standard for viral respiratory diagnosis. We have very powerful molecular tools, ensuring a quality respiratory viral diagnosis, available for all clinicians supporting hospitalized patients. One factor limiting this diagnosis is represented by the collected respiratory specimens. The main objectives of this work have been to study the cellularity of these clinical respiratory specimens, to propose a possible definition of what is commonly called “cellular richness,” and to measure the impact of this marker on the molecular viral diagnosis. Very few published studies have been completed in this area. However, a number of facts are commonly accepted within the medical community: respiratory specimen should be “rich” to allow for “good” viral diagnosis, the “good” samples are obtained almost exclusively in infants and children. In total we identified 7 studies published in international journals between 2003 and 2014, whose objective was to compare the various upper respiratory samples, in terms of sampling equipment (flocked swabs versus rayon swab), in terms of sampling site (nasal, oropharyngeal, nasopharyngeal, combining sites), and sampling modality (swab, wash, aspiration) [17–23].

The number of target cells and/or the number of extracellular viral particles collected could define the respiratory sample quality during sampling. It is not unreasonable to consider that the majority of the viral sequences detected by molecular techniques are mainly located in the intracellular compartment. However, several factors could modulate this distribution: the viral species, the cytopathic effect induced in vivo, the inflammatory responses, and the sampling delay from the onset of the symptoms. This study is retrospective; it was therefore not possible to collect data from the time between sampling and clinical symptoms in a standardized way. This matter is nonetheless very interesting and remains to be explored. For this study, we considered cell quantification, or cellular richness, as the main marker of diagnostic efficacy for a sample.

The 7 studies referenced above used indirect measurement of sample richness through the virus detected in it: 5 used molecular detection methods, and one was an antigen detection using rapid diagnostic test. The study published by Peter Daley et al. in 2006 is a direct immunofluorescence detection method. This method allows, when reading with a fluorescence microscope, to evaluate the number of infected and uninfected cells deposited on the slide [17]. Overall, the results are consistent and show a superiority of nasopharyngeal swab versus oropharyngeal and a superiority of flocked swab versus classical ones [14, 17, 19, 20]. The superiority of the “wash” versus the “swab” is not found by all authors, and comes well counteract the widespread idea that washing is always greater than swabbing [14].

Alsaleh et al. (2014) performed a molecular cell quantification using real time PCR in order to validate viral detection on nasal swabs. The cellularity of these samples was assessed using the quantification of a human endogenous retrovirus (ERV3) known to be present in two copies per diploid cell [24]. The authors report their results by comparing the Ct obtained upon ERV3 detection and not in terms of cellular load [18].

In our study, we made the choice to use a direct detection method for assessing the number of cells in respiratory samples. To the extent that we needed a large number of samples characterized by many markers for statistical analysis (age of the sampled patient, detected viral target, etc.), a prospective study on freshly sampled respiratory specimens could not be performed. We therefore used previously characterized and stored frozen (-80 °C) respiratory samples. The definition of cellular richness is not affected by freezing or thawing samples since it theoretically does not change the amount of HPRT1 nucleic acid, i.e. 2 copy per haploid cell, whether or not lysed. Similarly, no distinction between living and dead cells was made before the freezing process because such a distinction does not affect the quality of molecular detection by PCR (detection of viral genome into infected cells, living or not).

The analysis of 800 samples, divided into 400 positive and 400 negative in viral detection, failed to establish a minimum cellularity threshold to invalidate the negative results in viral molecular detection. The ROC curve shows that the cellularity is not a quantifiable predictor of the outcome of the virus detection.

However, our work has yielded interesting results concerning the factors influencing the cellularity of samples and the impact of cellularity on the result of molecular detection of respiratory viruses. We have clearly demonstrated that nasal aspirates allowed us to collect more cells than with nasal swabs and this only in the age group under 15 years old. This result must be tempered by the fact that 90 % (173/196) of the nasal aspirations of the study were from children under 9 years of age, since the distribution was random at inclusion of samples in the study. This reflects a common practice of sampling methods in clinical departments: nasal aspirates are rarely performed in adults and the elderly over 65 years old. This gesture is considered invasive and unpleasant. Our results support the idea that, de facto, it would not be appropriate to perform this type of sampling in this group.

For all types of samples (swabs and/or nasal aspirates), the age of the sampled patient remains an important marker influencing cellularity, with a maximum average cellularity under the age of 15, a minimum average cellularity in Adults group, and an intermediate average cellularity in the Elderly group. The reasons for this lack of cellularity in respiratory samples from Adults remain obscure.

Regarding the influence of cellularity on the result of viral detection, it should be noted that negative samples present, all age groups combined, an average cellularity lower than that obtained for the positive samples, even though it has not been possible to establish a predictive relationship. This difference was tenuous for the Children group. This observation leads to two possible explanations: either in the Positive group the infection increases the sample cellularity by promoting epithelial desquamation and, to a lesser extent, the mucus capture, containing free viral particles; or, in the Negative group, there are false-negatives in virus detection, induced by a lack of cells in the sample. This result was obtained from all samples (aspirates and swabs). Insofar as aspirates are evenly distributed in both Positive and Negative groups, we think that the result of comparison is not biased given that aspirates are richer samples. Considering viral detection in its entirety, without analysis of the viral species detected, it is interesting to note that the two factors, “cellularity” and “viral co-detection” (detection of 2 or 3 viruses) are associated positively. This suggests that the detection of several viruses is facilitated in the context of a rich sample. In cases of viral co-detection, the question of whether these are the same cells that are infected or not is not resolved, even if it is conventionally accepted that an already infected cell is less permissive to a second viral infection. It should also be noted that viral co-detection can be either a co-infection, or two or more sequential infections.

Such phenomena have already been discussed in the works of Alsaleh et al. which showed that the positive samples in viral detection had, on average, a greater amount of genetic material of human origin than negative ones. Similarly, samples where the gene ERV3 was not detected had lower viral detection. Finally, they also found that the positive samples for several viruses were also those in which cellular loads were highest [18].

The analysis of the potential impact of cellularity on the specific detection of various viruses included in the “respiratory panel” showed results that should be confirmed with larger numbers in each group. Indeed, on the one hand, the highest average cellularity was obtained in the hMPV positive samples, equally detected among Adults and Children groups; on the other hand, the lowest average cellularity is obtained in the RSV positive samples, mostly detected in Infants and Children groups. These results are surprising in that the two viruses, RSV and hMPV, belong to the same virus family (*Paramyxoviridae*) as well as to the same virus subfamily (*Pneumovirinae*), and are genetically close. They have many similarities in the circulatory mode and in the pathophysiology of the infection they cause. Yet the cellularity of hMPV positive samples is significantly greater than that of the RSV positive specimens.

## Conclusion

The quality of samples dramatically affects the quality of results provided to clinicians. It is important that a better understanding of the sample characteristics goes along with technological developments. This work is uncommon. He tries to give answers to a trivial scientific question: Respiratory specimens should they be « rich in cells » to ensure optimal virological molecular diagnosis? The cellular load is multifactorial and occurs for many in the sensitivity of molecular detection. However, it was not possible to determine a minimum threshold allowing molecular viral detection.

## Abbreviations

AdV, adenovirus; ARI, acute respiratory infection; CT, cycle threshold; DNA, deoxyribonucleic acid; EC, extraction control; ERV3, human endogenous retrovirus 3; Flu, influenzae virus; HBoV, human bocavirus; HCoV, human coronavirus; hMPV, human metapneumovirus; PCR, polymerase chain reaction; PIV, paraInfluenza virus; RhV/EV, rhino/entero virus; RNA, ribonucleic acid; ROC, receiver operating characteristic; RSV, human respiratory syncytial virus; SARS, severe acute respiratory syndrome; UTM, universal transport medium
